# Variação na detecção da síndrome congênita do Zika em função de alterações em protocolos

**DOI:** 10.26633/RPSP.2019.79

**Published:** 2019-09-20

**Authors:** Helaine Jacinta Salvador Mocelin, Thiago Nascimento do Prado, Paula de Souza Silva Freitas, Adelmo Inácio Bertolde, Freddy Perez, Lee W. Riley, Ethel Leonor Noia Maciel

**Affiliations:** 1 Universidade Federal do Espírito Santo (UFES) Laboratório de Epidemiologia (Lab-Epi) Vitória (ES) Brasil Universidade Federal do Espírito Santo (UFES), Laboratório de Epidemiologia (Lab-Epi), Vitória (ES), Brasil; 2 Universidade Federal do Espírito Santo (UFES) Departamento de Estatística Vitória (ES) Brasil Universidade Federal do Espírito Santo (UFES), Departamento de Estatística, Vitória (ES), Brasil.; 3 Organização Pan-Americana da Saúde (OPAS) Departamentos de Doenças Transmissíveis e Determinantes Ambientais da Saúde Washington (DC) Estados Unidos Organização Pan-Americana da Saúde (OPAS), Departamentos de Doenças Transmissíveis e Determinantes Ambientais da Saúde, Washington (DC), Estados Unidos.; 4 Universidade da Califórnia, Escola de Saúde Pública Divisão de Doenças Infecciosas e Vacinologia Berkeley (CA) Estados Unidos Universidade da Califórnia, Escola de Saúde Pública, Divisão de Doenças Infecciosas e Vacinologia, Berkeley (CA), Estados Unidos.

**Keywords:** Zika vírus, microcefalia, doenças transmissíveis, saúde pública, Brasil, Zika virus, microcephaly, communicable diseases, public health, Brazil, Virus Zika, microcefalia, enfermedades transmisibles, salud pública, Brasil

## Abstract

Em 2015, o Brasil enfrentou uma epidemia de infecção pelo vírus Zika que se propagou por países do mundo. Posteriormente, recomendações acerca dos critérios de notificação de casos de síndrome congênita do Zika (SCZ) foram divulgadas através de protocolos. As mudanças frequentes nessas recomendações podem ter afetado o gerenciamento clínico e o acesso a suporte pós-diagnóstico por crianças afetadas mas não identificadas. No presente estudo, 39 casos de SCZ notificados no estado do Espírito Santo no período de 2015 a 2016 foram reclassificados quanto ao seu diagnóstico de acordo com o protocolo atualmente vigente, diferente daquele que vigorava em 2015. Pela reclassificação, apenas oito dos 36 casos seriam confirmados, respeitando o critério de dois ou mais sinais ou sintomas da SCZ com ou sem microcefalia e confirmação sorológica. Ainda, pela diminuição no perímetro cefálico que define microcefalia, 17 casos passaram a não se enquadrar nessa condição. Apesar de o protocolo vigente não utilizar somente o perímetro cefálico como critério para notificação e confirmação da SCZ, cabe ressaltar que este achado ainda é o maior sinalizador para as equipes de saúde, indicando um risco da não detecção precoce da SCZ. Seria prudente uma revisão dos casos “descartados” no momento de transição entre protocolos, a fim de avaliar se foram corretamente classificados.

## Abstract (en)

In 2015, Brazil faced a Zika virus epidemic that spread to other countries in the world. As a result, recommendations regarding reporting criteria for congenital Zika syndrome (CZS) were issued in the form of protocols. The frequent changes in these recommendations may have affected clinical management and the access to post-diagnostic support by children who were affected by CZS, but who ended up not being identified. In the present study, 39 cases of CZS reported in the state of Espírito Santo, Brazil, from 2015 to 2016 were re-classified in terms of diagnosis using the current protocol, which is different from the protocol used in 2015. According to this re-classification, only eight out of 36 cases would be confirmed, based on the criterion of two or more signs or symptoms of CZS with or without microcephaly plus positive serologic results. Also, considering the decrease in the head circumference cut-off point defining microcephaly, 17 cases would no longer meet the definition for this condition. Even though the current protocol does not rely on head circumference alone for CZS reporting and confirmation, it should be noted that this is still the main sign considered by health care teams, and therefore the decrease in the cut-off point might have compromised early CZS detection. A review of “ruled out” cases would be advisable in moments of protocol transition to determine whether these cases have been correctly classified.

## RESUMEN

En el 2015, Brasil enfrentó una epidemia de infección por el virus del Zika que se propagó por varios países del mundo. Posteriormente, se divulgaron recomendaciones acerca de los criterios de notificación de casos del síndrome congénito por el virus del Zika (SCZ) por medio de protocolos. Los cambios frecuentes de esas recomendaciones podrían haber afectado el manejo clínico y el acceso al apoyo posterior al diagnóstico de los niños afectados, pero no identificados. En el presente estudio, se reclasificó el diagnóstico de 39 casos del SCZ notificados en el estado de Espírito Santo en el período 2015-2016, de acuerdo con el protocolo vigente en la actualidad, que es distinto del que regía en el 2015. Por causa de la reclasificación, se confirmaron únicamente ocho de los 36 casos, con observancia del criterio de dos o más signos o síntomas del SCZ acompañados o no de microcefalia y con confirmación serológica. Además, por la disminución del perímetro cefálico que define la microcefalia, 17 casos no correspondieron a esa afección. A pesar de que en el protocolo vigente no se utiliza solamente el perímetro cefálico como criterio para la notificación y confirmación del SCZ, cabe resaltar que este hallazgo es, con todo, la mayor señal para los equipos de salud, puesto que indica un riesgo de falta de detección temprana del SCZ. Convendría examinar los casos “descartados” en el momento de la transición entre protocolos, con el fin de determinar si se clasificaron correctamente.

Em 2015, o Brasil enfrentou uma epidemia de infecção pelo vírus Zika. Em novembro de 2015, o Ministério da Saúde declarou emergência nacional de saúde pública; em fevereiro de 2016, a Organização Mundial da Saúde (OMS) classificou a epidemia como emergência de saúde pública de interesse internacional. A partir de então, sucessivas recomendações acerca dos critérios de notificação da microcefalia fetal por vírus Zika foram publicadas através de protocolos e notas técnicas, com base em estudos que aconteciam em larga escala no país e no mundo (1, 2).

Enquanto estudiosos e pesquisadores estavam enfronhados na descoberta de novas informações sobre essa doença com manifestações singulares, a triagem de bebês no serviço de saúde utilizou, ao menos nos 2 anos iniciais da epidemia, o mesmo critério de identificação do primeiro caso no Nordeste brasileiro, em abril de 2015: presença ou não de microcefalia (3). Esse mesmo critério foi utilizado em serviços de saúde em todo o território, até mesmo quando já se sabia que o perímetro cefálico diminuído era apenas uma das manifestações de um quadro denominado síndrome congênita do Zika vírus (SCZ) – as crianças afetadas pela SCZ podem apresentar outras manifestações cerebrais além da redução do perímetro cefálico, que pode inclusive estar dentro dos limites normais. Outras manifestações dessa síndrome incluem danos oculares, problemas articulares, excesso de tônus muscular e convulsões, entre outros (4).

Por sua vez, o protocolo de vigilância da SCZ em vigor desde novembro de 2016 alterou a definição de microcefalia, mediante diminuição do perímetro cefálico que determina essa condição (5). Diante disso, acredita-se que possam existir crianças que nasceram com sequelas da SCZ, mas que, por não se enquadrarem na definição de perímetro cefálico diminuído, não recebem a assistência necessária. Em outras palavras, casos descartados na transição entre protocolos durante os primeiros 2 anos da epidemia podem de fato ser portadores de SCZ, estando sem acesso à rede assistencial e aos tratamentos necessários. Nesse sentido, o objetivo deste estudo foi descrever como as mudanças em protocolos relativos ao processo de investigação epidemiológica de casos de SCZ durante a epidemia podem ter afetado a detecção dessa doença.

## HISTÓRICO DOS PROTOCOLOS DE NOTIFICAÇÃO DE CASOS DE ZIKA VÍRUS

No Brasil, três protocolos oficiais para notificação de casos afetados por ZIKV foram divulgados desde 2015. O presente estudo analisou o protocolo de novembro de 2015 (Protocolo de vigilância e resposta à ocorrência de microcefalia relacionada à infecção do vírus Zika) e o último protocolo, lançado em novembro de 2016 e vigente até o momento (Orientações integradas de vigilância e atenção à saúde no âmbito da Emergência de Saúde Pública de Importância Nacional) (5, 6).

**FIGURA 1.**
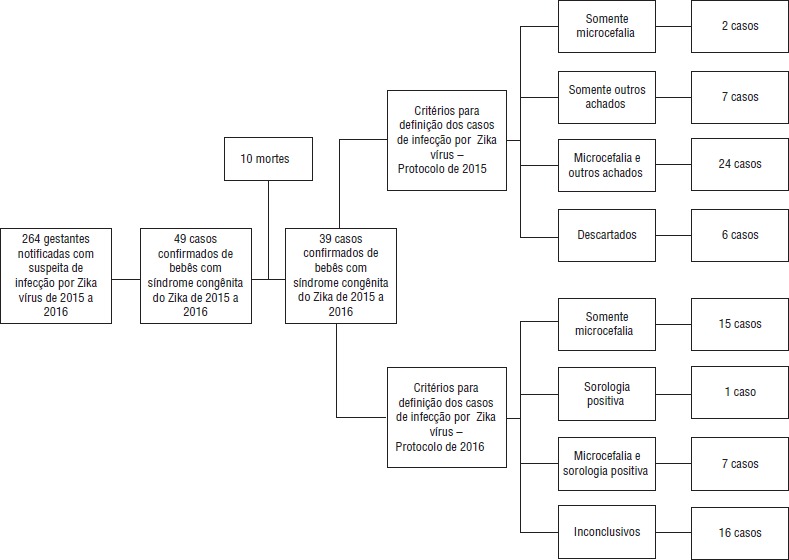
Definição dos casos de síndrome congênita de Zika detectados no estado do Espírito Santo, Brasil, em 2015 e 2016, de acordo com dois protocolos de notificação

A comparação entre o protocolo de 2015 e o protocolo vigente em 2019 mostra importantes diferenças, listadas na tabela 1. O protocolo de 2015 considerava como microcefalia um perímetro cefálico menor ou igual a 33 cm em recém-nascido vivo (menina ou menino) com 37 semanas ou mais de idade gestacional, conforme as referências da OMS (7). Além disso, o diagnóstico convencionado em 2015 para identificação de Zika vírus em tecido/fluido corporal de recém-nascido vivo ou mãe durante a gestação se dava pelo método de PCR até o 8º dia após a infecção pelo sangue, líquido cefalorraquidiano, urina ou lavagem brônquica. Esse diagnóstico precoce de PCR pode ter tido resultado falso negativo em muitos testes, uma vez que, no momento do exame, muitos casos suspeitos poderiam ter sido expostos fora desse período de detectabilidade viral, não sendo portanto rastreados para microcefalia fetal por Zika vírus (7). Em suma, no protocolo de 2015, era considerado caso confirmado de infecção por Zika vírus o bebê com perímetro cefálico menor que 33 cm e com PCR positivo da mãe ou do bebê (figura 1).

No protocolo que substituiu o de 2015, e que se encontra vigente no presente momento (2019), a microcefalia em recém-nascido com 37 semanas de gestação é definida por perímetro cefálico de 30,24 cm em meninas e 30,54 cm em meninos, adotando os parâmetros da tabela InterGrowth para ambos os sexos (http://www.saude.gov.br/saude-de-a-z/microcefalia/tabelas-da-oms-e-intergrowth) (8). Essa medida deve ser feita com a maior precisão possível (de preferência com precisão de dois decimais). Diante disso, considera-se caso confirmado de SCZ um recém-nascido com resultado positivo ou reativo para o vírus por sorologia até o 8º dia de vida e um resultado negativo ou inconclusivo em pelo menos um teste STORCH da mãe, durante a gravidez, ou do recém-nascido, e dois ou mais sinais e sintomas na imagem ou no exame clínico (podendo um deles ser a microcefalia fetal) (tabela 1) (9).

Quando se comparam os protocolos, nota-se que o de 2015 trata a microcefalia como o principal achado associado ao Zika vírus, enquanto o protocolo vigente, elaborado após as descobertas e avanços científicos sobre as malformações congênitas causadas pelo Zika vírus, trata da investigação epidemiológica de SCZ; essa investigação inclui ausência de sintomas ou de anormalidades aparentes no nascimento, ou seja, não se baseia somente na microcefalia (5, 6). Entretanto, o perímetro cefálico continua sendo o maior sinalizador de SCZ para a equipe de saúde.

## CLASSIFICAÇÃO DE CASOS CONFORME DOIS PROTOCOLOS DISTINTOS

Para ilustrar as diferenças entre os protocolos, será usado o exemplo do estado do Espírito Santo, onde, de janeiro de 2015 a dezembro de 2016, foram notificados 264 casos suspeitos de exposição ao Zika vírus em gestantes. Desses, 49 recém-nascidos foram confirmados com SCZ ou microcefalia fetal for Zika vírus (no início da epidemia, não existia o termo SCZ) de acordo com o protocolo de 2015. Dos 49 recém-nascidos, 10 morreram, permanecendo na amostra 39 crianças (10).

O levantamento dos casos confirmados de crianças com SCZ no estado baseou-se em dados de notificações de prontuários epidemiológicos da Secretaria de Saúde do Estado (SESA) no período de 1º de janeiro de 2015 a 31 de dezembro de 2016. Este levantamento foi parte de um projeto de pesquisa mais amplo, aprovado pelo Comitê de Ética em Pesquisa do Centro de Ciências da Saúde da Universidade Federal do Espírito Santo (CEP/CCS/UFES, parecer 1 730 231 de 16/09/2016). Também foi aprovado pelo Comitê de Ética Internacional (PAHOERC) sob o parecer PAHO-2017-02-0013. Os dados foram trabalhados somente pelos autores do manuscrito e o banco de dados permaneceu em computador codificado. Resultados recentes desse projeto descreveram o perfil de 25 das 39 mães das crianças incluídas na amostra (11), revelando mulheres majoritariamente pretas e pardas, com mais de 30 anos. Das mulheres que trabalhavam antes de ter o bebê com SCZ, 75% foram demitidas ou forçadas a pedir demissão. A maioria residia em áreas de periferia e em condições precárias, morando de aluguel. Mais da metade das famílias tinha renda domiciliar *per capita* de até 249,96 reais (61,72 dólares) e não recebia o benefício de prestação continuada (BPC) – conferido pelo sistema de assistência social brasileiro às famílias de pessoas com deficiência – no valor de, aproximadamente, um salário mínimo mensal (que equivale a aproximadamente R$ 953,16 ou US$ 235,35 conforme taxa de câmbio em 21 de setembro de 2018). Esses dados sugerem a pobreza como um determinante social na configuração da epidemia do Zika vírus no país (11). Estudos realizados no Nordeste reforçam essa sugestão de pobreza como um determinante social, descrevendo um perfil semelhante ao descrito acima: mulheres com baixa escolaridade, pardas ou negras e pobres, vivendo em bairros mais desfavorecidos e em condições precárias de vida (9, 12).

**TABELA 1.** Critérios para confirmação de infecção por Zika vírus em recém-nascidos de acordo com protocolo de 2015 e com protocolo vigente, Brasil, 2019

Critério	Protocolo 2015	Protocolo vigente em 2019
Perímetro cefálico (cm)		
Menino	33cm	30,54cm
Menina	33cm	30,24cm
Critério diagnóstico	PCR positivo da mãe ou do recém-nascido ou microcefalia diagnosticada por método de imagem e excluídas outras possíveis causas	PCR positivo do recém-nascido + Storch negativo + sinais e sintomas
Caso confirmado	Perímetro cefálico ≤ 33cm PCR positivo da mãe ou do bebê	Perímetro cefálico ≤ 30,54 cm para meninos ou 30,24 cm para meninas + PCR positivo do bebê; e/ou Storch negativo ou inconclusivo da mãe ou do recém-nascido e dois ou mais dos sinais e sintomas, como calcificações cerebrais, desproporção craniofacial, alteração do tônus muscular na imagem ou no exame clínico

Como mostra a figura 1, utilizando como referência o protocolo de 2015 (6), 26 (66,66%) crianças da amostra do Espírito Santo seriam confirmadas com base em microcefalia (somente microcefalia ou microcefalia com outros achados, como PCR ou exames de imagens). Além disso, sete (17,94%) crianças seriam confirmadas somente por outros achados, sem microcefalia fetal, e seis (15,38%) crianças seriam descartadas para a doença. Ou seja, de acordo com esse protocolo, 33 crianças seriam consideradas como portadoras de SCZ.

Já com base no protocolo vigente em 2019 (5), 22 (55,41%) crianças teriam microcefalia, sendo 15 (38,46%) com achado de microcefalia sem resultado de sorologia para Zika vírus (por provável falha no processo de investigação epidemiológica) e sete (17,94%) com microcefalia somada a sorologia positiva com STORCH negativo ou inconclusivo. Além disso, uma (2,56%) criança foi confirmada com dois ou mais achados neurológicos e sorologia positiva para Zika vírus sem microcefalia fetal. Dezesseis (41,02%) seriam consideradas como casos prováveis, com investigação epidemiológica inconclusiva, que ocorre quando não é possível realizar a investigação etiológica por motivo de recusa ou por não ser possível encontrar a criança após três tentativas, se os resultados e informações disponíveis não permitem classificar a criança em outra categoria (figura 1).

Dessa forma, conforme o protocolo vigente, somente oito (20,51%) crianças seriam confirmadas com SCZ, por terem dois ou mais sinais ou sintomas da referida síndrome, incluída ou não a microcefalia fetal, juntamente com a confirmação sorológica. Além disso, dos 39 casos confirmados no estado apenas com base em microcefalia, 17 (35%) não apresentaram microcefalia de acordo com o protocolo vigente. No entanto, esses 17 recém-nascidos possuem manifestação clínica da SCZ.

## DISCUSSÃO

As mudanças no protocolo de notificação de casos de Zika vírus geraram dificuldades no relato de casos no Estado do Espírito Santo. Os testes sorológicos utilizados para confirmar a SCZ conforme o protocolo atual não eram rotina no início da epidemia. Os casos que carecem de resultados de confirmação com base no protocolo mais recente são classificados como “provável/inconclusivo” e continuam a ser seguidos e avaliados para o desenvolvimento cognitivo e motor da criança, visto que atrasos no marco de desenvolvimento infantil tornam-se evidentes somente durante estágios posteriores, como perda de audição ou diminuição da acuidade visual (4).

Nesse sentido, deve-se ponderar sobre a importância de monitorar as crianças nascidas durante os surtos, treinar os profissionais de saúde para identificar os atrasos no desenvolvimento neurocognitivo infantil e aprimorar a qualidade do pré-natal em busca de sinais e sintomas do ZIKV na gestação. O rastreio durante toda a gestação é justificável ao analisar que, em 18 (37%) das mães apresentadas neste estudo e que tiveram infecção confirmada, não foi detectada doença exantemática na gestação. Isso impõe uma dificuldade adicional no rastreio da SCZ, uma vez que os sintomas da infecção pelo Zika vírus não são percebidos pela gestante. Por isso, seria necessária a testagem para o Zika vírus como rotina nas gestantes de regiões que possuem casos da doença.

Ainda quanto às limitações da implementação do protocolo vigente, cabe ressaltar que há uma barreira importante de aplicabilidade da nova metodologia de vigilância. Por exemplo, a precisão exigida na medição do perímetro cefálico é uma limitação, uma vez que não se encontra disponível nos serviços de saúde do país instrumento de aferição com duas casas decimais. Ainda, os demais critérios de inclusão não são de fácil interpretação, exceto para profissionais familiarizados com ortopedia pediátrica ou neurologia pediátrica. Quanto à detecção de malformação congênita, há uma expectativa de que sejam investigados os casos com alterações nas imagens de ultrassonografia realizadas na gravidez. Contudo, as alterações são mais visíveis no terceiro trimestre da gestação e, como o serviço público de saúde brasileiro normalmente só garante uma ultrassonografia durante toda a gestação, dificilmente esse será um critério de inclusão (4).

Por fim, dado o conhecimento atual dos efeitos do vírus Zika no desenvolvimento das crianças, e dada a maior abrangência do primeiro protocolo no que se refere à microcefalia, o mais prudente seria uma revisão dos casos “descartados” no momento de transição entre protocolos, a fim de avaliar se seriam casos prováveis ou confirmados por outras especialidades, tais como otorrinolaringologista ou oftalmologia. Seria ainda importante acompanhar o crescimento e o desenvolvimento dessas crianças. Finalmente, faz-se necessário avaliar se o protocolo vigente é compatível com um sistema de saúde com fragilidades e dificuldades de uniformização de condutas, como ocorre no Brasil.

### Contribuição dos autores.

HJSM, TNO, PSSF e ELNM, conceberam a ideia original e desenharam a pesquisa. AIB, FP e LWR obtiveram os dados e redigiram o artigo. Todos os autores analisaram e interpretaram os dados, revisaram criticamente o conteúdo e revisaram e aprovaram a versão final.

### Agradecimentos.

Este trabalho é parte do projeto “As políticas públicas em situações de emergência: análise do surto de Zika vírus”, um produto da dissertação de mestrado de HJSM. Agradecemos à Secretaria de Saúde do estado do Espírito Santo pela solicitude em repassar os dados e às mulheres diretamente afetadas pelo surto.

### Financiamento.

Este estudo foi financiado pela Organização Pan-Americana da Saúde (OPAS) por meio do edital Programa conjunto HRP/TDR/OPS de *Pequeñas subvenciones a la investigación sobre el brote del virus de Zika en las Américas*.

### Declaração.

As opiniões expressas no manuscrito são de responsabilidade exclusiva dos autores e não refletem necessariamente a opinião ou política da RPSP/PAJPH ou da Organização Pan-Americana da Saúde (OPAS).
